# Rab5 is critical for SNAP23 regulated granule-granule fusion during compound exocytosis

**DOI:** 10.1038/s41598-017-15047-8

**Published:** 2017-11-10

**Authors:** Ofir Klein, Amit Roded, Neta Zur, Nurit P. Azouz, Olga Pasternak, Koret Hirschberg, Ilan Hammel, Paul A. Roche, Ayaka Yatsu, Mitsunori Fukuda, Stephen J. Galli, Ronit Sagi-Eisenberg

**Affiliations:** 1 Department of Cell and Developmental Biology, Sackler Faculty of Medicine, Tel Aviv University, Tel Aviv, 69978 Israel; 2 Department of Pathology, Sackler Faculty of Medicine, Tel Aviv University, Tel Aviv, 69978 Israel; 3 Experimental Immunology Branch, National Cancer Institute, National Institutes of Health, Bethesda, Maryland 20892 USA; 4 Laboratory of Membrane Trafficking Mechanisms, Department of Developmental Biology and Neurosciences, Graduate School of Life Sciences, Tohoku University, Aobayama, Aoba-ku, Sendai, Miyagi 980-8578 Japan; 5 Departments of Pathology and of Microbiology and Immunology, and Sean N. Parker Center for Allergy and Asthma Research, Stanford University School of Medicine, Stanford, California, 94305-5176 USA; 6 Present Address: Division of Allergy and Immunology, Cincinnati Children’s Hospital Medical Center, University of Cincinnati, Cincinnati, Ohio USA

## Abstract

Compound exocytosis is considered the most massive mode of exocytosis, during which the membranes of secretory granules (SGs) fuse with each other to form a channel through which the entire contents of their granules is released. The underlying mechanisms of compound exocytosis remain largely unresolved. Here we show that the small GTPase Rab5, a known regulator of endocytosis, is pivotal for compound exocytosis in mast cells. Silencing of Rab5 shifts receptor-triggered secretion from a compound to a full exocytosis mode, in which SGs individually fuse with the plasma membrane. Moreover, we show that Rab5 is essential for FcεRI-triggered association of the SNARE protein SNAP23 with the SGs. Direct evidence is provided for SNAP23 involvement in homotypic SG fusion that occurs in the activated cells. Finally, we show that this fusion event is prevented by inhibition of the IKKβ2 kinase, however, neither a phosphorylation-deficient nor a phosphomimetic mutant of SNAP23 can mediate homotypic SG fusion in triggered cells. Taken together our findings identify Rab5 as a heretofore-unrecognized regulator of compound exocytosis that is essential for SNAP23-mediated granule-granule fusion. Our results also implicate phosphorylation cycles in controlling SNAP23 SNARE function in homotypic SG fusion.

## Introduction

Regulated exocytosis is a key mechanism for intercellular communication and also contributes to host defenses against environmental challenges. Depending on the type of trigger, exocytosis may occur *via* full fusion (i.e., of single secretory granules [SGs] with the plasma membrane), kiss-and-run transient fusion, or compound exocytosis. The latter involves homotypic fusion of SGs prior or sequential to SG fusion with the plasma membrane thereby enabling the discharge of the contents of SGs that are located at intracellular locations distal to the plasma membrane surface. Compound exocytosis is therefore considered the most extensive mode of cargo release^1^. Compound exocytosis has been documented in both exocrine and endocrine cells^2–8^ and in immune cells including eosinophils^9–11^ and neutrophils^12^, where rapid discharge of mediators is required to kill invading pathogens such as parasites or bacteria, and mast cells^13,14^, where the efficient release of pre-stored inflammatory mediators contributes both to innate immune responses^15,16^ and to allergic reactions and anaphylaxis^17–19^.

Despite the physiological importance of compound exocytosis, the precise molecular mechanisms that underlie this process have remained poorly resolved^1,2,20,21^. Indeed, one of the major challenges faced in this regard is to differentiate, based on functional assays, the fusion machinery that mediates SG fusion with the plasma membrane from the fusion machinery involved in homotypic granule-granule fusion. Two SNARE proteins have been implicated in mediating SG fusion during compound exocytosis. Studies in pancreatic acinar cells have demonstrated the involvement of VAMP8^2,20^. By contrast, SNAP25 and its close homolog SNAP23 have been strongly implicated, though not directly proven, in playing a role in this process on the basis of their redistribution from the plasma membrane to the SGs during compound exocytosis in pancreatic β cells and mast cells, respectively^13,22^. In mast cells, knockdown of SNAP23 reduced FcεRI-stimulated secretion by 30%^23,24^, whereas redistribution from the plasma membrane to SGs occurred in permeabilized cells into which calcium and GTPγS had been introduced, conditions that parallel stimulated compound exocytosis^13^. However, these results do not identify the exact step that is regulated by this SNARE. Indeed, the opposing effects exerted by SNAP23 on granule fusion with the plasma membrane in pancreatic exocrine and endocrine secretion^25^, taken together with the well documented involvement of SNAP23 in multiple cellular processes, including the fusion of recycling endosomes with the plasma membrane^26^, raises the possibilities that SNAP23 either may impact exocytosis indirectly, by affecting endocytic recycling which then influences exocytosis^27,28^, or may contribute to exocytosis directly, by enhancing or inhibiting SG fusion with the plasma membrane and/or mediating granule–granule fusion during compound exocytosis.

To answer this question, we have established an experimental model that allows us to directly visualize homotypic granule fusion. Following up on our previous work, which identified the small GTPase Rab5 as regulator of the granule-granule fusion that occurs during the biogenesis of mast cell SGs^29^, we took advantage of the fact that giant SGs are formed in mast cells that express constitutively active (CA) Rab5 mutants^29^. These giant SGs, which preserve their exocytosis competence^29^, are easy to visualize and quantify by digital microscopy and therefore offer excellent opportunities for addressing directly the mechanism of granule–granule fusion that occurs during compound exocytosis. Here, we used this experimental paradigm to seek direct evidence of the involvement of SNAP23 in mediating homotypic SG fusion during compound exocytosis. Furthermore, given the important role of Rab5 in regulating SG fusion during their biogenesis, we also explored the intriguing possibility that Rab5 might be involved in regulating receptor-triggered SG fusion during compound exocytosis. Here we provide evidence that SNAP23 stimulates the granule-granule fusion that occurs in mast cells in response to antigen (Ag)-induced crosslinking of cell-bound IgE, conditions that activate the FcεRI and trigger compound exocytosis. We also demonstrate the importance of IKKβ2-mediated phosphorylation of SNAP23, as well as SNAP23 dephosphorylation, in regulating SNAP23 function. Finally, we identify for the first time a pivotal role for Rab5 in FcεRI receptor-stimulated SG fusion, namely, Rab5-dependent regulation of SNAP23 targeting to the SGs.

## Results

### SNAP23 stimulates receptor-triggered homotypic SG fusion but has no impact on SG fusion with the plasma membrane

**Figure 1:**
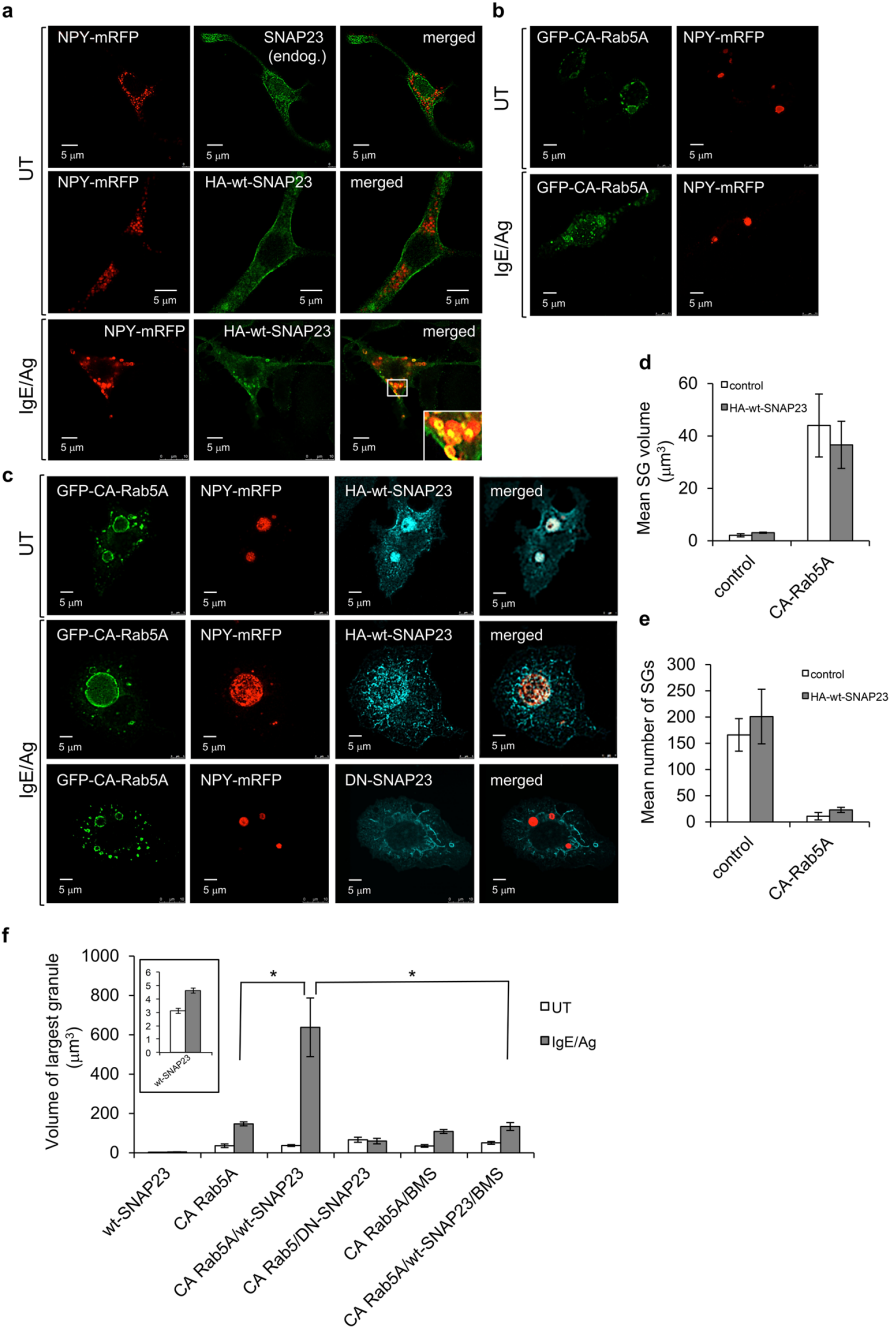
SNAP23 promotes formation of super giant SGs in IgE/Ag triggered cells. **(a**–**c)** RBL cells were co-transfected with 10 μg NPY-mRFP and 20 μg of either empty pcDNA3 vector (these cells would express only endogenous SNAP23) or HA-wt-SNAP23, or triple-transfected with 10 μg NPY-mRFP, 15 μg of pEGFP-CA Rab5A and 20 μg of either empty pcDNA3 vector in (**b**) or HA-wt-SNAP23 or DN-SNAP23 in (**c**), as indicated. Cells were either left untreated (UT) or sensitized with 1 μg/ml of IgE. Twenty-four hours after transfection, cells were either left untreated (UT) or triggered with 50 ng/ml of DNP-HSA (Ag) for 10 min, as indicated. Cells were fixed and immunostained using rabbit polyclonal antibodies directed against SNAP23 or monoclonal antibodies directed against HA followed by Hilyte Plus 647-conjugated goat anti-mouse IgG. Cells were analyzed by confocal microscopy. Bars = 5 μm. The inset in (**a**) is the enlargement of the boxed area showing co-localization between SNAP23 and NPY-mRFP. **(d,e**) The volumes and number of SGs in 20 untreated cells (i.e., in the absence of stimulation with IgE and DNP-HSA) were calculated from confocal images by the Imaris software. The mean volume of a SG and the mean number of SGs per cell are presented. Data are means ± SEM. **(f)** The volume of NPY-mRFP containing granules with largest diameter determined using the Imaris software was calculated. The mean volume of the largest SGs in 20 cells from each treatment is presented. Data are means ± SEM *P < 0.01 (unpaired two-tailed Student’s *t*-test). The inset is the enlargement of the graph showing the volume of the largest SGs in cells expressing HA-wt-SNAP23 alone.

Vesicle fusion depends on the precise coupling of SNARE proteins that zipper into stable membrane-bridging (“trans”) complexes^30^. Therefore, overexpression of a relevant SNARE may either disturb the fusion process, if in excess, or enhance fusion in case it is rate limiting^31^. In accord with this model, we recently demonstrated the involvement of VAMP8 in the Rab5-stimulated fusion of SGs that occurs during SG biogenesis^29^. Taking the same approach, we here analyzed the impact of overexpression of SNAP23 on SG size in CA Rab5-expressing cells. To this end, we first confirmed the correct targeting of overexpressed HA-tagged wild type SNAP23 (HA-wt-SNAP23) in RBL-2H3 mast cells. Consistent with previous reports by others and us, both the endogenous and overexpressed wt-SNAP23 predominantly localized to the plasma membrane, but were also detected at intracellular structures, presumably endosomes^13,32–35^ (Fig. 1a). However, unlike VAMP8, whose overexpression abrogated the formation of giant SGs by CA Rab5A^29^, overexpression of SNAP23 alone (Fig. 1a [panels labeled HA-wt-SNAP23]) or together with CA Rab5A (Fig. 1c compared with 1b) had no demonstrable impact on the ability of expression of CA Rab5A to increase SG size (3.1 ± 0.2 *vs* 2.1 ± 0.6 μm^3^ in the absence of CA Rab5A and 36.6 ± 9 *vs* 44 ± 12 μm^3^ in CA Rab5A expressing cells), as detected by the co-expression of NPY-mRFP, the SG reporter^29,36,37^ (Fig. 1d), or decrease SG number (166 ± 31 *vs* 201 ± 52 in the absence of CA Rab5 and 11 ± 7 *vs* 23 ± 5 in CA Rab5A expressing cells) (Fig. 1e). Hence, overexpression of SNAP23 neither inhibited nor did it enhance the Rab5-regulated SG fusion that occurs during SG biogenesis (Fig. 1d,e). Therefore, these results strongly suggest that, similarly to its close homolog SNAP25^38^, SNAP23 plays no role in the homotypic fusion of the SGs that occurs during their biogenesis.

In sharp contrast, a dramatic difference in SG structure was noted when cells co-expressing CA Rab5A and HA-wt-SNAP23 were subjected to an IgE/antigen (Ag) trigger (Fig. 1c). Morphological analysis of the triggered cells revealed the appearance of ‘super giant’ SGs (Fig. 1c), whose average size was significantly larger (an increase of ~ 400%) than the size of the largest granule detected in IgE/Ag-triggered cells expressing CA Rab5A but not HA-wt-SNAP23 (Fig. 1b,f). Formation of ‘super giant’ SGs required the co-expression of CA Rab5A and SNAP23 and was not detected in triggered cells that overexpressed only CA Rab5A (Fig. 1b,f) or HA-wt-SNAP23 (Fig. 1a,f). To exclude the possibility that the increased volume of the SGs was the result of impaired fusion of multi-vesicular SGs with the plasma membrane, we analyzed the impact of HA-wt-SNAP23 overexpression on secretion. We found that overexpression of HA-wt-SNAP23 alone, or together with CA Rab5A, did not inhibit the secretion of granule content, as monitored by measuring the release of NPY-mRFP (see Supplementary Fig. S1). These results support the conclusion that SG fusion with the plasma membrane is normal in such cells, thus strongly implicating SNAP23 in mediating receptor-triggered SG fusion with each other before or during their fusion with the plasma membrane, the hallmark of compound exocytosis. Consistent with this notion, overexpression of the dominant-negative, carboxy terminal-truncated mutant of SNAP23^33^ failed to enhance SG enlargement observed in CA Rab5A expressing cells in response to an IgE/Ag trigger (Fig. 1c,f).

### IκappaB kinase (IKKβ)2-mediated phosphorylation of SNAP23 is essential for IgE/Ag-triggered homotypic SG fusion

**Figure 2:**
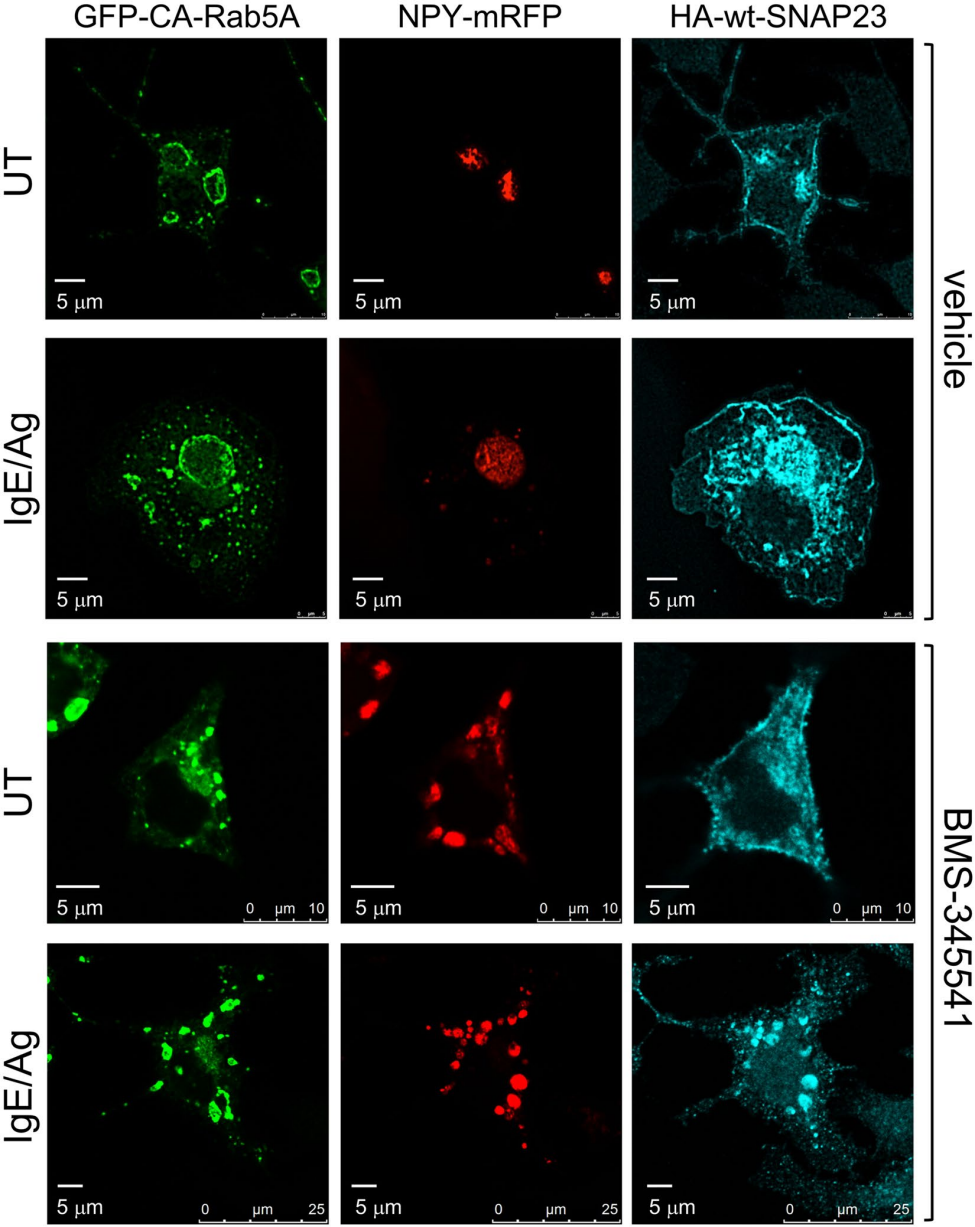
Inhibition of IKKβ2 prevents SNAP23 promoted formation of super giant SGs in IgE/Ag triggered cells. RBL cells were triple-transfected with 10 μg NPY-mRFP, 15 μg of pEGFP-CA Rab5A and 20 μg of HA-wt-SNAP23. Cells were either left untreated (UT) or sensitized with 1 μg/ml of IgE. Twenty-four hours after transfection, cells were pre incubated with BMS-345541 (5 μM) or vehicle and further incubated for 10 min without or with 50 ng/ml DNP-HSA (Ag) as indicated. Cells were fixed and immunostained using monoclonal antibodies directed against HA followed by Hilyte Plus 647-conjugated goat anti-mouse IgG. Cells were analyzed by confocal microscopy. Bars = 5 μm.

SNAP23 undergoes phosphorylation in IgE/Ag-triggered RBL cells^39^ and IKKβ2 was identified as the responsible kinase^24^. However, the precise role of this kinase in mast cell degranulation is not completely resolved^24,40^. We recently showed that pharmacological inhibition of IKKβ2 by BMS-345541 can influence the pattern of IgE/Ag-triggered mast cell degranulation, including reducing the mean size of exteriorized granule structures, suggesting a role for this kinase in compound exocytosis^19^. To confirm this notion, we analyzed whether IKKβ2 inhibition influenced the capacity of SNAP23 to support formation of super giant SGs in IgE/Ag-triggered, CA Rab5A-expressing cells. We found that exposure of the co-transfected cells to BMS-345541 prior to cell triggering with Ag completely prevented the formation of the super giant SGs (Fig. 2). Quantitative analysis of the results revealed that, in the presence of BMS-345541, the size of the largest SGs in IgE/Ag-triggered cells was the same in the absence or presence of wild type SNAP23 overexpression, revealing the failure of HA-wt-SNAP23 overexpression to further increase the size of SGs in response to IgE/Ag-triggering in IKKβ2-inhibited cells (Fig. 1f [CA Rab5A/BMS panels]). Notably, at the same range of concentrations, BMS-345541 had little or no effect on IgE/Ag-triggered secretion, as assessed by release of the endogenous granule-stored mediator, β-hexosaminidase (see Supplementary Fig. S1). These results indicate that IKKβ2-mediated phosphorylation is important for granule-granule fusion, but not for granule fusion with the plasma membrane.

### Phosphorylation cycles of SNAP23 are essential for homotypic SG fusion during compound exocytosis

**Figure 3:**
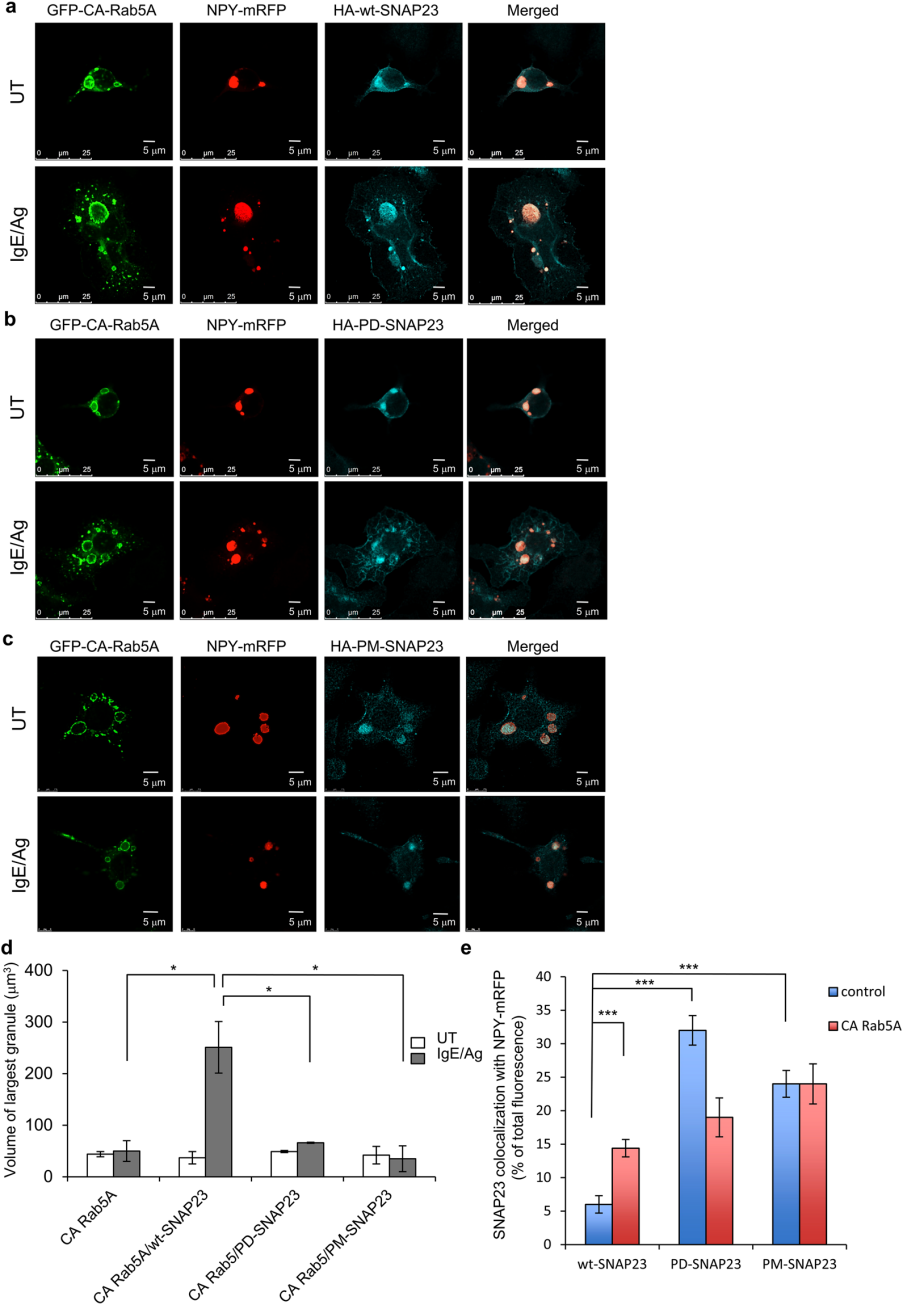
SNAP23 phosphorylation mutants fail to support formation of super giant SGs. **(a**–**c**) RBL cells were triple-transfected with 10 μg NPY-mRFP, 15 μg of pEGFP-CA Rab5A and 20 μg of either HA-wt-SNAP23, or HA-PD-SNAP23 or HA-PM-SNAP23, as indicated. Cells were either left untreated (UT) or sensitized with 1 μg/ml of IgE. Twenty-four hours after transfection, cells were either left untreated (UT) or triggered with 50 ng/ml of DNP-HSA (Ag) for 10 min. Cells were fixed and immunostained using monoclonal antibodies directed against HA followed by Hilyte Plus 647-conjugated goat anti-mouse IgG. Cells were analyzed by confocal microscopy. Bars = 5 μm. (**d**) The size of the largest NPY-mRFP containing granules was calculated using the Imaris software. The mean volume of the largest SG in each of 15 cells from each treatment group is presented. Data are means ± SEM *P < 0.05 (unpaired two-tailed Student’s *t*-test). (**e**) RBL cells were triple-transfected with 10 μg NPY-mRFP and 15 μg of either pEGFP (control) or pEGFP-CA Rab5A and 20 μg of either HA-wt-SNAP23, or HA-PD-SNAP23 or HA-PM-SNAP23, as indicated. Co-localization of SNAP23 (wt, PD or PM) with NPY-mRFP in control or CA Rab5A expressing cells was quantified by the Imaris software. The data are means ± SEM derived from four independent experiments. ***P (wt-SNAP23 control vs. wt-SNAP23 CA Rab5A) = 4.4 × 10^−5^, n = 40 for control cells and 17 for CA Rab5A expressing cells; ***P (wt-SNAP23 control vs. PD-SNAP23 control) = 2.5 × 10^−9^, n = 30 for PD-SNAP23 cells; ***P (wt-SNAP23 control vs. PM-SNAP23 control) = 3 × 10^−9^, n = 19 for PM-SNAP23 cells (unpaired two-tailed Student’s *t*-test).

IKKβ2 was shown to phosphorylate SNAP23 on serine residues Ser 95 and Ser 120 in IgE/Ag-triggered RBL cells^24,39^. To verify that IKKβ2-mediated phosphorylation of SNAP23 is required for granule-granule fusion, we next analyzed the capacity of a phosphorylation-deficient mutant of SNAP23, in which serine 95 and serine 120 were substituted by alanine [i.e., SNAP23(S95A/S120A), herein referred to as PD-SNAP23], to support formation of super giant SGs. These experiments demonstrated that, in sharp contrast to wt-SNAP23 (HA-wt-SNAP23 in Fig. 3a and also Figs 1c and 2), co-expression of PD-SNAP23 with CA Rab5A failed to generate super giant SGs in IgE/Ag-triggered cells (Fig. 3b). Quantitative analysis of the results indicated that the size of the largest SGs in such cells remained essentially the same as in cells expressing CA Rab5A alone (Fig. 3d).

Because SNAP23 is phosphorylated by IKKβ2 in an IgE/Ag-dependent fashion^24,39^, we anticipated that a phosphomimetic mutant of SNAP23 might bypass the need for cell activation and would enhance SG fusion to form super giant SGs even in resting cells. However, contrary to that expectation, we found that overexpression of a SNAP23 double mutant, in which serine 95 and serine 120 were replaced by glutamic acid to mimic the negative charge of the phosphorylated SNARE protein [SNAP23(S95E/S120E), herein referred to as PM-SNAP23], failed to increase the SG size in resting cells beyond their size in cells expressing CA Rab5A alone (Fig. 3c,d). Furthermore, overexpression of this phosphomimetic mutant also failed to support formation of super giant SGs in IgE/Ag-triggered cells (Fig. 3c,d). Notably, overexpression of neither the phosphorylation-deficient mutant (PD-SNAP23) nor the phosphomimetic mutant (PM-SNAP23) detectably affected IgE/Ag-stimulated exocytosis (see Supplementary Fig. S1). These results suggest that both IKKβ2-mediated phosphorylation of SNAP23 and SNAP23 dephosphorylation are required for trigger-dependent SG fusion, although we cannot exclude the possibility that the introduced mutations in PM-SNAP23 fail to fully recapitulate its genuine phosphorylated state.

### Phosphorylation controls the cellular distribution of SNAP23, but not SG-targeting in response to an IgE/Ag trigger

**Figure 4:**
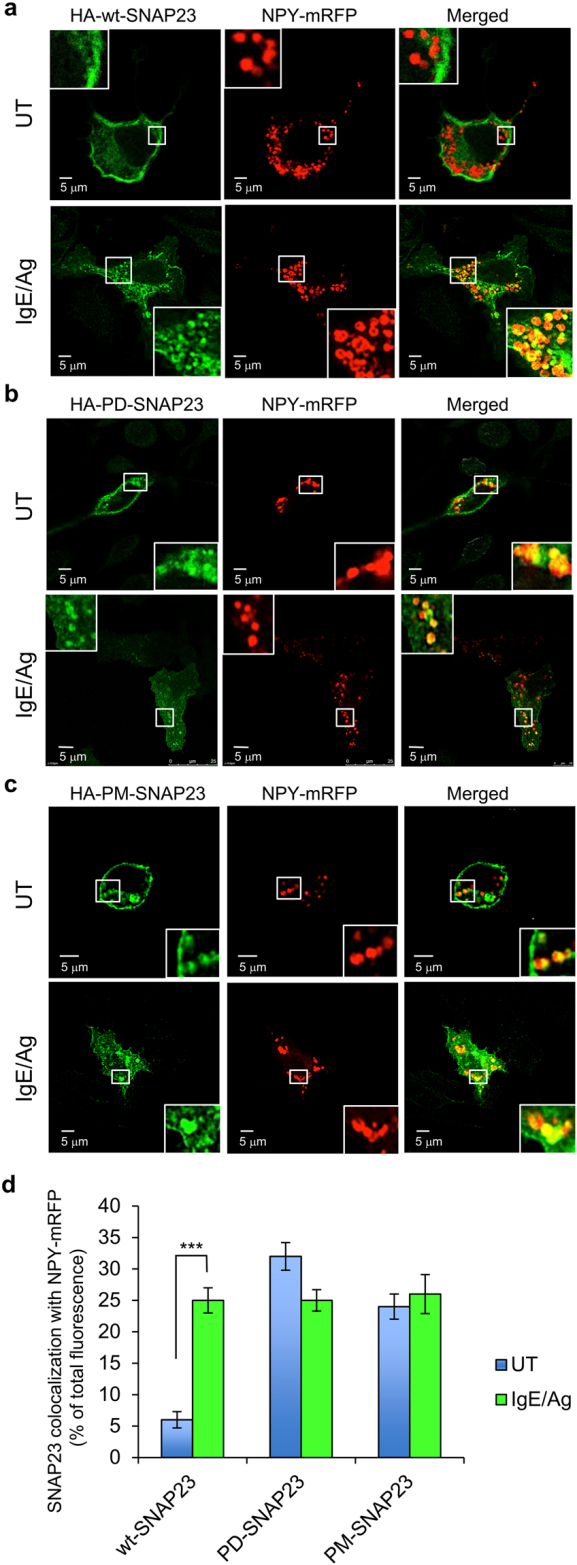
SNAP23 redistributes from the plasma membrane to the SGs in IgE/Ag-triggered cells. (**a**–**c**) RBL cells were co-transfected with 15 μg NPY-mRFP and 30 μg of either HA-wt-SNAP23, or HA-PD-SNAP23, or HA-PM-SNAP23, as indicated. Cells were either left untreated (UT) or sensitized with 1 μg/ml of IgE. Twenty-four hours after transfection, cells were either left untreated (UT) or triggered with 50 ng/ml of DNP-HSA (Ag) for 10 min. Cells were fixed and immunostained using monoclonal antibodies directed against HA followed by Hilyte Plus 488-conjugated goat anti-mouse IgG. Cells were analyzed by confocal microscopy. Bars = 5 μm. The insets are enlargements of the boxed areas. **(d)** Co-localization of SNAP23 (wt, PD or PM) with NPY-mRFP in non-triggered (UT, see also Fig. 3e) or IgE/Ag-triggered cells, was quantified by the Imaris software. The data are means ± SEM derived from four independent experiments. ***P (UT wt-SNAP23 vs. IgE/Ag wt-SNAP23) = 1.5 × 10^−10^, n = 40 cells for each condition (unpaired two-tailed Student’s *t*-test).

SNAP23 was reported to reside at the plasma membrane in resting mast cells and to relocate from the plasma membrane to the SGs in permeabilized cells into which GTPγS, that activates GTP binding proteins, was introduced^13^. Consistent with this result, we noticed that when co-expressed with CA Rab5A, SG location of HA-wt-SNAP23 became more prominent even in the absence of cell trigger (compare Fig. 1c [top row] and 3a [top row] with Fig. 1a [middle row]). Quantification of the results demonstrated a ~2-fold increase in SNAP23 co-localization with NPY-mRFP in CA Rab5A co-expressing cells (Fig. 3e). This finding prompted us to investigate the route of trafficking of SNAP23 in intact, IgE/Ag-triggered mast cells. These experiments revealed a significant (~4 fold) increase in co-localization between HA-wt-SNAP23 and NPY-mRFP in response to the IgE/Ag trigger (Fig. 4a,d and also Fig. 1a [middle and lower rows]), thus demonstrating trigger-dependent association of HA-wt-SNAP23 with the SGs in intact, physiologically-triggered, mast cells. Intriguingly, analysis of the cellular distribution of SNAP23 phosphorylation mutants revealed that both the phosphorylation-deficient and the phosphomimetic mutants displayed a more biased distribution towards intracellular SG locations either with or without the IgE/Ag trigger (Fig. 4b–d) or CA Rab5A co-expression (Fig. 3b,c and e). These findings indicate that over-expression of these SNAP23 mutants influences the cellular distribution of SNAP23 under basal conditions (i.e., it increases the association of SNAP23 with the SGs in unstimulated cells), but triggering the cells with IgE and Ag results in little or no additional association of these mutant forms of SNAP23 with the SGs.

### Rab5 is required for SG targeting of SNAP23 in IgE/Ag-triggered cells

**Figure 5:**
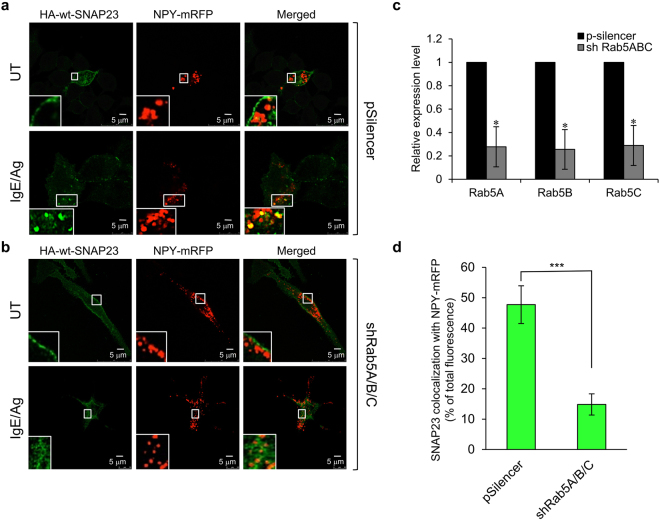
SG targeting of SNAP23 is Rab5-dependent. (**a**) RBL cells were co-transfected with 15 μg NPY-mRFP, 15 μg of HA-wt-SNAP23 and 30 μg of pSilencer. **(b)** RBL cells were co-transfected with 15 μg NPY-mRFP, 15 μg of HA-wt-SNAP23 and 15 μg of shRab5A and 15 μg of shRab5B/C. Cells were either left untreated (UT) or sensitized with 0.5 μg/ml of IgE. Forty-eight hours after transfection, cells were either left untreated (UT) or triggered with 50 ng/ml of DNP-HSA (Ag) for 10 min. Cells were fixed and immunostained using monoclonal antibodies directed against HA followed by Hilyte Plus 488-conjugated goat anti-mouse IgG. Cells were analyzed by confocal microscopy. Bars = 5 μm. The insets are enlargements of the boxed areas. **(c)** Relative expression of Rab5A/B/C mRNA in pSilencer- versus shRab5A/B/C-expressing cells, prior to their IgE/Ag trigger (i.e. UT cells) was determined by real-time PCR. (**d**) Co-localization of SNAP23 with NPY-mRFP in the IgE/Ag triggered cells was quantified by the Imaris software. The data are derived from the analysis of a total of 19 cells from 3 separate experiments, ***P < 0.001 (unpaired two-tailed Student’s *t*-test).

The finding that SNAP23 localizes to the SGs in unstimulated cells that express an active Rab5 mutant prompted us to explore the possibility that Rab5 regulates SG-targeting of SNAP23. To test this, we characterized the localization of HA-wt-SNAP23 in resting and IgE/Ag-triggered cells in which Rab5 was knocked down. Using Rab5A/B/C-targeting shRNAs, which we previously showed can effectively knockdown the Rab5 isoforms that are endogenously expressed in RBL cells^29^, we found that, in cells not stimulated with IgE/Ag, HA-wt-SNAP23 localized predominantly to the plasma membrane in cells transfected with either control shRNA (Fig. 5a [pSilencer]) or Rab5-targeting shRNAs (Fig. 5b [shRab5A/B/C]), in which Rab5 mRNA expression was reduced by ~70% (Fig. 5c). As expected, HA-wt-SNAP23 redistributed to the SGs in response to an IgE/Ag trigger in control, pSilencer-treated, cells (Fig. 5a), displaying significant co-localization with NPY-mRFP (Fig. 5d). However, careful examination of the Rab5A/B/C-knockdown cells stimulated with IgE/Ag revealed that HA-wt-SNAP23 translocated to intracellular tubular structures that were in close proximity, yet distinct from the NPY-mRFP containing SGs (Fig. 5b). Moreover, quantitative analysis of the results revealed an ~70% reduction in the extent of wt-SNAP23 co-localization with NPY-mRFP after IgE/Ag stimulation in the Rab5A/B/C knockdown cells (Fig. 5d).

### Rab5 is pivotal for compound exocytosis

**Figure 6:**
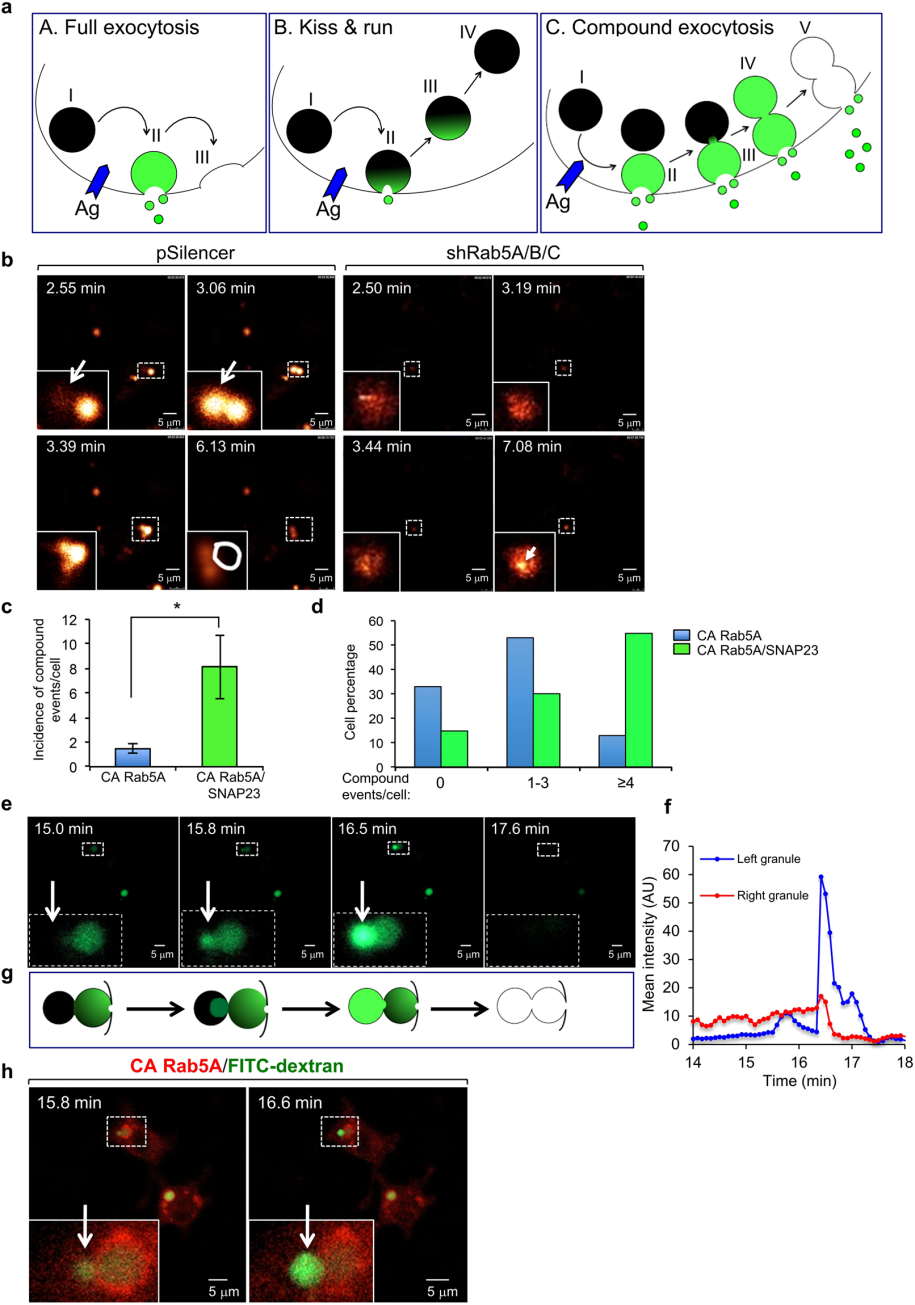
Rab5 is essential for IgE/Ag-triggered compound exocytosis. **(a)** A scheme illustrating the distinct modes of exocytosis and how they are measured by the de-quenching of endocytosed FITC-dextran. Cells are incubated with FITC-dextran, which is incorporated into the SGs. However, because of the acidic pH of the SGs, the fluorescence of FITC is quenched and the granule has no fluorescence (shown here as a black color) (A–C, I). When IgE sensitized cells are triggered to degranulate by specific antigen (Ag), the FITC fluorescence signal changes as a function of the mode of exocytosis. During full exocytosis, formation of a fusion pore between the single SG and the plasma membrane allows efflux of protons leading to the alkalization of the SG lumen. As a result, the fluorescence of FITC de-quenches and the SG becomes fluorescent (shown here as a green color) (A, II). This results in a short (less than 5 sec) burst of fluorescence that rapidly dissipates as the granule membrane fully merges with the plasma membrane and the contained FITC-dextran is released and diluted in the external medium (A, III). During kiss-and-run exocytosis, again, formation of the fusion pore leads to alkalization of the SG and de-quenching of the intra-granular FITC (shown here as an area of green color) (B, II). However, since the SG rapidly detaches and re-acidifies, FITC fluorescence quenches and the event is reflected in a small and short-lived fluorescence change (B, III- IV). Compound exocytosis starts, like the full exocytic event, with a fluorescent burst that corresponds to the alkalization of the single SG that fused with the plasma membrane (C, II). However, rather than emptying quickly and losing fluorescence upon release of the FITC probe, the SG, that is still connected to the membrane via a stable fusion pore, fuses with another SG, whose quenched fluorescence then de-quenches as it gains access to the external milieu and alkalinizes (C, III-IV). This results in a larger and significantly longer-lived burst of fluorescence (C, IV) than is seen in the other forms of degranulation, before fluorescence is lost due to the release of the FITC probe from the fused SGs (C, V). Therefore, while both full exocytosis and kiss-and-run exocytosis are associated with short, small bursts of fluorescence, compound exocytosis is reflected by the appearance of bursts of large and sustained fluorescent structures. Occasionally, the fusion event itself can be detected by the gradual de-quenching of a SG that is adjacent to and has fused with a fluorescent, hence exocytosing, SG (C, III). (**b**) RBL cells were co-transfected with 15 μg NPY-mRFP and either 30 μg of pSilencer or 15 μg of shRab5A, and 15 μg of shRab5B/C, as indicated. Cells were sensitized with 1 μg/ml of IgE after loading with FITC-dextran (1 mg/ml) for 48 h. Cells were triggered by 50 ng/ml of DNP-HSA (Ag) and visualized by time-lapse fluorescence microscopy as described in the Materials and Methods. The images corresponding to the indicated time periods after triggering with Ag are from Supplementary Videos 1 (**b**, pSilencer) and 5 (**b**, shRab5A/B/C) on line. The white arrow and white circle in (**b**, pSilencer) indicate transient bursts of large yellow fluorescent structures, indicative of exteriorization of fused SGs. The white arrow in (**b**, shRab5A/B/C) points to tiny fluorescent structures that persisted in Rab5-knockdown cells, likely to be indicative of single granules being exteriorized without compound fusion. Of note, the intensity in this image was increased to allow detection of the signal. (**c**–**h**) RBL cells were co-transfected with 10 μg of mStr-CA Rab5A and 20 μg of either empty vector (CA Rab5A) or HA-wt-SNAP23 (CA Rab5A/SNAP23). Cells were sensitized with 1 μg/ml of IgE after loading with FITC-dextran (1 mg/ml) for 48 h. Cells were triggered by 50 ng/ml of DNP-HSA (Ag) and visualized by time-lapse fluorescence microscopy as described in the Materials and Methods. (**c**) The average number of compound exocytosis events/cell was determined based on data derived from 4 separate videos for each transfection and a total of 15 cells for CA Rab5A and 13 cells for CA Rab5A/SNAP23. *P = 0.026 (unpaired two-tailed Student’s *t*-test). (**d**) The percentage of cells that failed to develop any compound exocytosis events, or developed 1–3 events/cell or developed 4 or more compound exocytosis events/cell was calculated. (**e**) Images captured at the designated time points after triggering with Ag (presented in Supplementary Video 4), demonstrate the de-quenching of FITC-dextran fluorescence (shown in green) during the fusion between a quenched SG (left, shown in black) and an adjacent SG (right, shown in green) that already fused with the plasma membrane and is therefore de-quenched and visible. The insets are the enlargements of the boxed areas. The white arrows point to the position of the SG that is in the process of de-quenching as it merges with the exocytosing, already de-quenched SG. (**f**) Quantification of the fluorescence changes during the fusion process. The right SG displays a constant level of fluorescence for approximately four minutes (13–17 min), implying that it is connected to the plasma membrane and therefore de-quenched and is in the process of releasing cargo until cargo discharge is complete at time point 17:6 min. The left SG is initially quenched but displays a 6–10 fold increase in FITC fluorescence after merging with the exocytosing SG. FITC fluorescence then drops upon the release of the cargo. (**g**) A schematic presentation of the process of homotypic SG fusion that is shown in (**e**). (**h**) Presentation of both the red and green channels of the designated time points derived from Video 4, showing the position of mSTR-CA Rab5A (red) around the fusing SGs that contain FITC-dextran (green, when de-quenched).

The identification of a Rab5 requirement for SG targeting of wt-SNAP23 strongly suggested that Rab5, a known regulator of endocytic pathways^41^, is pivotal for compound exocytosis. To test this intriguing idea, we took advantage of the fact that monitoring the fluorescence of fluorescein isothiocyanate (FITC)–dextran-loaded SGs can permit imaging of exocytic events in mast cells^14,19^. Specifically, exposure of the SG interior contents to the external milieu, as a result of opening of a fusion pore between the SG membrane and the plasma membrane, increases the pH of the SG, which in turn de-quenches the fluorescence of the pH-sensitive FITC fluorophore. Therefore, a small burst of fluorescence corresponds to the full fusion of a SG with the plasma membrane, and this event is transient because of the rapid dissipation of the signal as the probe diffuses away in the external medium. A small burst of fluorescence that quenches slowly corresponds to kiss-and-run exocytosis, when the probe persists in the retrieved, alkalinized SG until re-acidification takes place. However, more persistent bursts of large fluorescent structures reflect compound exocytosis of SGs that fused sequentially around the time of formation of the fusion pore, which enables the de-quenching of their fluorescence prior to their final discharge^14,19^ (Fig. 6a).

In IgE/Ag-triggered RBL cells, full exocytosis of single SGs is the primary mode of exocytosis, but kiss and run and sequential compound exocytosis have been noted as well^14^. In agreement with these results, monitoring FITC fluorescence in control cells revealed numerous tiny bursts of fluorescence indicative of full exocytosis events (see Video 1). However, this pattern then gradually changed, giving rise to the appearance of large, yet transient, fluorescent structures, consistent with the pattern of compound exocytosis (Fig. 6b [pSilencer] and Supplementary Video 1). Analysis of the live cell images revealed the appearance of a FITC fluorescence burst, indicative of granule fusion with plasma membrane that was immediately followed by the de-quenching of a second granule that merged with the first one, consistent with an event of sequential granule fusion followed by the discharge of the probe (Fig. 6b [pSilencer]). The development of compound exocytosis was associated with the appearance of aggregates of NPY-mRFP in the cells as well as with increased fluorescence of FITC in the medium, reflecting the release of the probe (Supplementary Video 2 and Fig. S2). Enlargement of the SGs by the expression of CA Rab5A attenuated the appearance of the large fluorescent bursts, as compared to non-transfected cells present in same field (Supplementary Video 3). This observation is consistent with the idea that a limiting factor, namely SNAP23, restricted the compound fusion of the enlarged SGs. Indeed, co-transfection with CA Rab5A and HA-wt-SNAP23, conditions that led to the formation of super giant SGs in triggered cells, markedly increased the number of fluorescent events (Supplementary Video 4). Quantitative analysis of the results demonstrated that the average number of large fluorescence bursts, indicative of compound exocytosis events, increased from 1.5 events/cell in IgE/Ag triggered cells that expressed CA Rab5A alone, to 8.1 events/cell in triggered cells that co-expressed CA Rab5A and SNAP23 (Fig. 6c). Furthermore, while 33% of the cells transfected with CA Rab5A exhibited no evidence of compound exocytosis, 53% developed one to three compound exocytosis events, and only 14% exhibited more than 4 events, by contrast, 55% of the cells that expressed CA Rab5A and SNAP23 developed more than four compound exocytosis events/cell (Fig. 6d). Careful inspection of the live cell images enabled us to identify the sequential fusion of SGs that was reflected in the gradual de-quenching of a previously quenched granule that was adjacent to a bright granule (Fig. 6e and f). The de-quenching of the latter SGs implies that they already fused with the plasma membrane, prior to fusing with the newly de-quenching, more internally located, SGs. These findings are fully in accord with the changes that would be expected to occur during the homotypic SG fusion which is characteristic of sequential compound exocytosis (see scheme Fig. 6g). Importantly, examination of the position of CA Rab5A demonstrated its presence on the fusing granule consistent with its critical role in the fusion process (Fig. 6h).

In sharp contrast, the pattern of exocytosis did not change in Rab5A/B/C knockdown cells triggered with IgE/Ag, in which tiny bursts of fluorescence persisted with no evidence for sequential granule fusion or appearance of either aggregates of NPY-mRFP within the cells or release of the FITC probe to the medium, most likely due to its rapid dissipation in the medium when released in small quanta (Fig. 6b [shRab5A/B/C] and Supplementary Videos 5 and 6 and Fig. S3). The lack of signal was not due to a defect in FITC-dextran loading because addition of ammonium chloride, which alkalinizes the SGs, caused an immediate increase in FITC-dextran fluorescence confirming the proper loading of the probe (Supplementary Video 6 and Fig. S3).

## Discussion

A role for SNAP23 has been implicated in compound exocytosis based on its redistribution from the plasma membrane to SGs, with kinetics paralleling that of compound exocytosis^13^. However, the direct involvement of SNAP23 in mediating granule-granule fusion has not previously been demonstrated. Taking advantage of the fact that expression of a constitutively-active mutant of Rab5 in mast cells stimulates SG fusion, thereby generating giant SGs that maintain their exocytosis competence^29^, we used morphometric analysis to investigate the role of SNAP23 in granule-granule (i.e., SG-SG) fusion. This approach overcomes the difficulty in interpreting the results of functional assays of secretion that are influenced by the involvement of SNAP23 in cellular processes that may impact exocytosis indirectly. Here we show that SNAP23 stimulates formation of super giant SGs in activated mast cells. Because fusion of SGs with the plasma membrane is not impaired, these super giant SGs are likely the product of multi-granular fusion that occurred exclusively in IgE/Ag-triggered cells, thus consistent with homotypic SG fusion during compound exocytosis. Indeed, we provide direct evidence for the stimulation of compound exocytosis by overexpression of HA-wt-SNAP23 in CA Rab5A-expressing cells, using live cell imaging to monitor the de-quenching of loaded FITC-dextran, a method previously used by others and us to distinguish the different modes of mast cell exocytosis^14,19^. In documenting a delay in the development of compound exocytosis upon IgE/Ag triggering, we also demonstrated the sequential nature of the granule fusion event. This finding is consistent with the exaggerated and also variable size of the super giant SGs that likely reflects both multi-granular fusion and also swelling of the granule matrix that occurs following fusion of the granule membrane with the plasma membrane.

Being able to monitor trigger-stimulated granule fusion directly allowed us to analyze the role of IKKβ2-mediated phosphorylation of SNAP23 in this process. Functional assays have demonstrated a variable contribution of IKKβ2 to secretion^19,24,40^. In our hands, pharmacological inhibition of IKKβ2 did not affect secretion but abrogated the formation of the super giant SGs in activated cells. Furthermore, SNAP23 mutants that affected those sites which are phosphorylated by IKKβ2 also failed to support formation of super giant SGs. Our finding that both phosphodeficient and phosphomimetic mutants are non-functional in this assay suggests that dynamic cycling between phosphorylated and dephosphorylated states is required to accomplish this function. This notion is highly compatible with previous data that documented low levels of SNAP23 phosphorylation in IgE/Ag-activated cells, and the transient nature of such phosphorylation^39^. Taken together, our results strongly suggest that while IKKβ2-mediated phosphorylation of SNAP23 is dispensable for granule fusion with the plasma membrane, it is crucial for compound exocytosis, although we cannot exclude the possibility that fusion between SGs might also be mediated by yet unknown proteins that are regulated by IKKβ2, besides or in addition to SNAP23. The contribution of IKKβ2 to overall secretion thus might vary in conjunction with the effects of other factors that influence the contribution of compound exocytosis to overall secretion. Further support for this notion comes from the findings that knockout of IKKβ2 or RNAi of SNAP23 had partial and relatively modest impacts on secretion^23,24^.

Which factor determines the extent of compound exocytosis? We show that single overexpression of HA-wt-SNAP23 does not increase SG fusion in triggered cells and that compound exocytosis occurs also in the absence of HA-wt-SNAP23 overexpression. Therefore, SNAP23 is not rate limiting under normal conditions. However, SNAP23 becomes the limiting factor in the presence of hyperactive Rab5, conditions under which overexpression of HA-wt-SNAP23 allows further SG fusion and the extent of compound exocytosis is increased. We therefore propose that Rab5 is a key regulator that is pivotal for compound exocytosis. Indeed, we show that RNAi of Rab5 largely abrogates compound exocytosis in our model. At least one mechanism by which Rab5 may regulate compound exocytosis is by mediating SNAP23 targeting to the SGs. Given our previous findings that Rab5 mediates endosome fusion with the SGs^29^, SG targeting of SNAP23 might be accomplished by Rab5 regulated fusion between SNAP23-associated endosomes and the SGs. According to this model, SG targeting of SNAP23 occurs in two steps: the first involves trigger-dependent association of SNAP23 with lipid rafts and syntaxin 4^34^, followed by dissociation from the plasma membrane and association with endosomes; whereas the second step involves Rab5-dependent fusion of SNAP23-associated endosomes with the SGs.

The precise mechanisms of plasma membrane targeting and internalization of SNAP23 are presently unknown. Palmitoylation^42^, binding to distinct syntaxins^42,43^ and electrostatic interactions^35^ have been implicated in these processes. We show that SNAP23 phosphorylation mutants display enhanced SG binding even in non-triggered cells. Thus, it is possible that phosphorylation/dephosphorylation may impact palmitoylation, binding to syntaxins, or protein charge, thereby affecting the cellular distribution of SNAP23 under basal conditions. Internalization of SNAP25, the close homolog of SNAP23, was shown to depend on Arf6^44^. Whether or not SNAP23 follows a similar route is presently unknown, but our future studies will address these questions.

Notably, although SNAP23 targeting to the SGs might be a mechanism by which Rab5 regulates compound exocytosis, this is not necessarily the only mechanism. Our results implicating sequential SG fusion in mediating IgE/Ag-triggered compound exocytosis implies that stable fusion pores may be needed in this process. The latter are critical to allow the SGs that are fusing with the plasma membrane to serve as hook for the attachment of subsequent fusing SGs. Because the stability of the fusion pore positively correlates with the diameter of the exocytosing vesicle^45^, it is possible that Rab5 may additionally control compound exocytosis by increasing the size of SGs during their biogenesis^29^. According to this model, active Rab5 dictates the relative contribution of compound to overall exocytosis both before mast cell stimulation, by determining the size distribution of the cells’ SGs via regulation of their homotypic fusion, and after IgE/Ag stimulation, by mediating SG fusion with SNAP23-containing endosomes. Finally, Rab5 might directly control granule-granule fusion in analogy to its function in endosome fusion^46^ or SG fusion during their biogenesis^29^.

How the level of Rab5 activation is regulated is presently unknown and awaits future investigation. In this context, it is interesting to note that GTPγS has long been recognized as a critical factor for compound exocytosis, implicating the involvement of GTP-binding proteins in this process^47^. Our results are in full accord with this notion, and provide several lines of evidence that Rab5 represents a pivotal GTP-binding protein involved in compound exocytosis.

In summary, we have used CA Rab5A-expressing mast cells that display giant, exocytosis-competent SGs, to analyze the granule-granule fusion that occurs in triggered mast cells during compound exocytosis. This approach allowed us to demonstrate for the first time evidence for a direct role of SNAP23 and SNAP23 phosphorylation cycles in FcεRI-triggered homotypic SG fusion. Importantly, we also identified Rab5 as a novel regulator of compound exocytosis and provide mechanistic insights into its mode of action. Finally, our results introduce an experimental model system that can be used for further analyses of the fusion machinery that mediates granule-granule fusion during compound exocytosis.

## Methods

### Antibodies and Reagents

Monoclonal mouse anti-DNP IgE (Cat #D8406, clone SPE-7), DNP-human serum albumin (HSA), FITC-dextran (150 kDa) and BMS-345541 were from Sigma-Aldrich (St. Louis, MO). Hilyte Plus 647-conjugated goat anti-mouse IgG (Cat # AS-61057-05-H647) and Hilyte Plus 488-conjugated goat anti-mouse IgG (Cat # 61057-H488) were from Anaspec (Fremont, CA). HRP-conjugated goat anti-mouse (Cat # 115-035-166) and anti-rabbit (Cat # 115-035-003) IgG were from Jackson Immuno Research Laboratories (West Grove, PA). Monoclonal anti-HA (MMS-101R, clone 16B12) was from Covance (Princeton, NJ). Rabbit polyclonal anti SNAP23 antibodies were from Abcam (Cat # ab4114).

### Plasmids used in this study

pEGFP-C1-CA Rab5A was prepared as previously described^48^. NPY-mRFP was a gift from Dr. U. Ashery (Tel Aviv University, Tel Aviv, Israel). DN-SNAP23 was previously described^33^. The open reading frame of mouse SNAP23^36^ was subcloned into the pEF-HA vector^49^. pEF-HA-SNAP23(S95E/120E) and pEF-HA-SNAP23(S95A/120 A) were constructed by using the following mutagenic oligonucleotides (mutated nucleotides are underlined): 5′-AACTTTGAGGCTGGAAAGAA-3′ (SNAP23-S95 A sense), TTCTTTCCAGCCTCAAAGTT-3′ (SNAP23-S95A antisense), 5′-AAGCAACCGGCCCGGATTAC-3′ (SNAP23-S120A sense), 5′-GTAATCCGGGCCGGTTGCTT-3′ (SNAP23-S120A antisense), 5′-AACTTTGAGGAAGGAAAGAA-3′ (SNAP23-S95E sense), TTCTTTCCTTCCTCAAAGTT-3′ (SNAP23-S95E antisense), 5′-AAGCAACCGGAACGGATTAC-3′ (SNAP23-S120E sense), and 5′-GTAATCCGTTCCGGTTGCTT-3′ (SNAP23-S120E antisense). Rab5A/B/C shRNA and the control pSilencer vector were previously described^29^.

### Cell Culture

RBL-2H3 cells were maintained in adherent cultures in DMEM supplemented with 10% FBS and 100 μg/ml streptomycin and 100 units/ml penicillin in a humidified atmosphere of 5% CO_2_ at 37 °C.

### Transient transfection

Transient transfection of RBL cells was performed as described previously^36^. Briefly, RBL cells (1.5 × 10^7^) were transfected by electroporation at 300 V and pulse length of 20 mSec, using an ECM830 electroporator (BTX, USA). The cells were immediately replated in tissue culture dishes containing growth medium for the desired time periods.

### Immunostaining and confocal analyses

RBL cells (4 × 10^5^ cells/ml) were grown on 12-mm round glass cover-slips and incubated overnight with mouse anti-DNP specific monoclonal IgE. Following three washes in Tyrode buffer (10 mM HEPES [pH 7.4], 130 mM NaCl, 5 mM KCl, 1.8 mM CaCl_2_, 1 mM MgCl_2_, 5.6 mM glucose, and 0.1% BSA), cells were stimulated in same buffer at 37 °C with 50 ng/ml of DNP-HSA. Cells were subsequently washed three times with PBS and fixed for 20 min at room temperature with 4% paraformaldehyde in PBS. Cells were then permeabilized for 30 min at room temperature with 0.1% Triton X-100, 5% FBS, and 2% BSA diluted in PBS. Cells were subsequently incubated for 1 h at room temperature with the primary Abs, followed by three washes and a 1-h incubation with the appropriate secondary Abs. After washing, cells were mounted (Golden Bridge Life Science, Mukilteo City, WA) and analyzed using a LEICA STED high resolution laser scanning confocal microscope (Leica, Wetzlar, Germany) and a 63 oil/1.4 numerical aperture objective. Quantification of SGs size and extent of co-localization was carried out by Imaris software (Bitplane, Zurich, Switzerland).

### Rab5 silencing

RBL cells (1.5 × 10^7^ cells/ml) were transiently transfected with control pSilencer vector or with shRNAs directed against Rab5A and Rab5BC, as previously described^29^. Forty-eight hours later, total RNA was purified using the PerfectPure RNA purification system (5 Prime) according to the manufacturer’s instructions, and the expression levels of the Rab5 isoforms was determined by quantitative RT-PCR (ABI Prism 7900 SDS, Applied Biosystems), using a SYBR Green real-time PCR Kit (Applied Biosystems) as previously described^29^. The following primers were used: Rab5A, reverse primer 5′-TGT GCA GGC TCA GTA AGG TC-3′, forward primer 5′-GCT AAG ACA TCA ATG AAT GTA AAT GAA-3′; Rab5B, reverse primer 5′-CTG CATATG CCT GAG CCT CT-3′, forward primer 5′-ACA AAG CTG ACC TTG CCA AC-3′; Rab5C, reverse primer 5′-CAA ACT TGA CCG TTG TGT CG-3′, forward primer 5′-CCA GGA GAG CAC AAT TGG A-3′.

### Time-lapse microscopy of living cells

RBL cells were seeded at 2 × 10^5^ cells/chamber in eight-well chamber borosilicate coverglass systems (Thermo Fisher Scientific Waltham, MA). Cells were loaded with 1 mg/ml of FITC-dextran for 48 h. Images were acquired by a Leica Sp5 laser scanning confocal microscope, equipped with a heated chamber (37 °C) and CO_2_ controller (4.8%) and a C-Apochromat 363/1.2 W Corr objective or using a Zeiss LSM Pascal confocal microscope using a Plan-apochromat 63° – NA 1.4 objective (Carl Zeiss MicroImaging) or Zeiss LSM 800 microscope. Image capture was performed using the standard time-series option (Carl Zeiss MicroImaging). Images and videos were generated and analyzed using the Zeiss LSM software and ImageJ software (W. Rasband, National Institutes of Health, Bethesda, MD). Long time-lapse image sequences were captured using the autofocusing function.

### Statistical analysis

Data are expressed as means ± SEM. The p values were determined by an unpaired two-tailed Student’s *t* test.

## Electronic supplementary material

Supplementary Information

Video 1

Video 2

Video 3

Video 4

Video 5

Video 6
