# Supplementary material for: Rab5 is critical for SNAP23 regulated granule-granule fusion during compound exocytosis
Source: Sci Rep. 2017 Nov 10;7:15315. doi: 10.1038/s41598-017-15047-8 (PMC5681557; doi:10.1038/s41598-017-15047-8)
Supplement: Supplementary file 1 — Supplementary Information [file 41598_2017_15047_MOESM1_ESM.pdf]

Supplementary Information for

Rab5 is critical for SNAP23 regulated granule-granule fusion during compound exocytosis.

Ofir Klein[1+], Amit Roded[1+], Neta Zur[1+], Nurit P Azouz[1++], Olga Pasternak[1], Koret Hirschberg[2], Ilan Hammel[2], Paul A Roche[3], Ayaka Yatsu[4], Mitsunori Fukuda[4], Stephen J Galli[5] and Ronit Sagi-Eisenberg[1*]

[1]Department of Cell and Developmental Biology, Sackler Faculty of Medicine, Tel Aviv University, Tel Aviv 69978, Israel, [2]Department of Pathology, Sackler Faculty of Medicine, Tel Aviv University, Tel Aviv 69978, Israel [3]Experimental Immunology Branch, National Cancer Institute, National Institutes of Health, Bethesda, Maryland, 20892, USA, [4]Laboratory of Membrane Trafficking Mechanisms, Department of Developmental Biology and Neurosciences, Graduate School of Life Sciences, Tohoku University, Aobayama, Aoba-ku, Sendai, Miyagi 980-8578, Japan, [5]Departments of Pathology and of Microbiology and Immunology, and Sean N. Parker Center for Allergy and Asthma Research, Stanford University School of Medicine, Stanford, California, 94305-5176, USA.

+These authors contributed equally to this work.
*Corresponding author

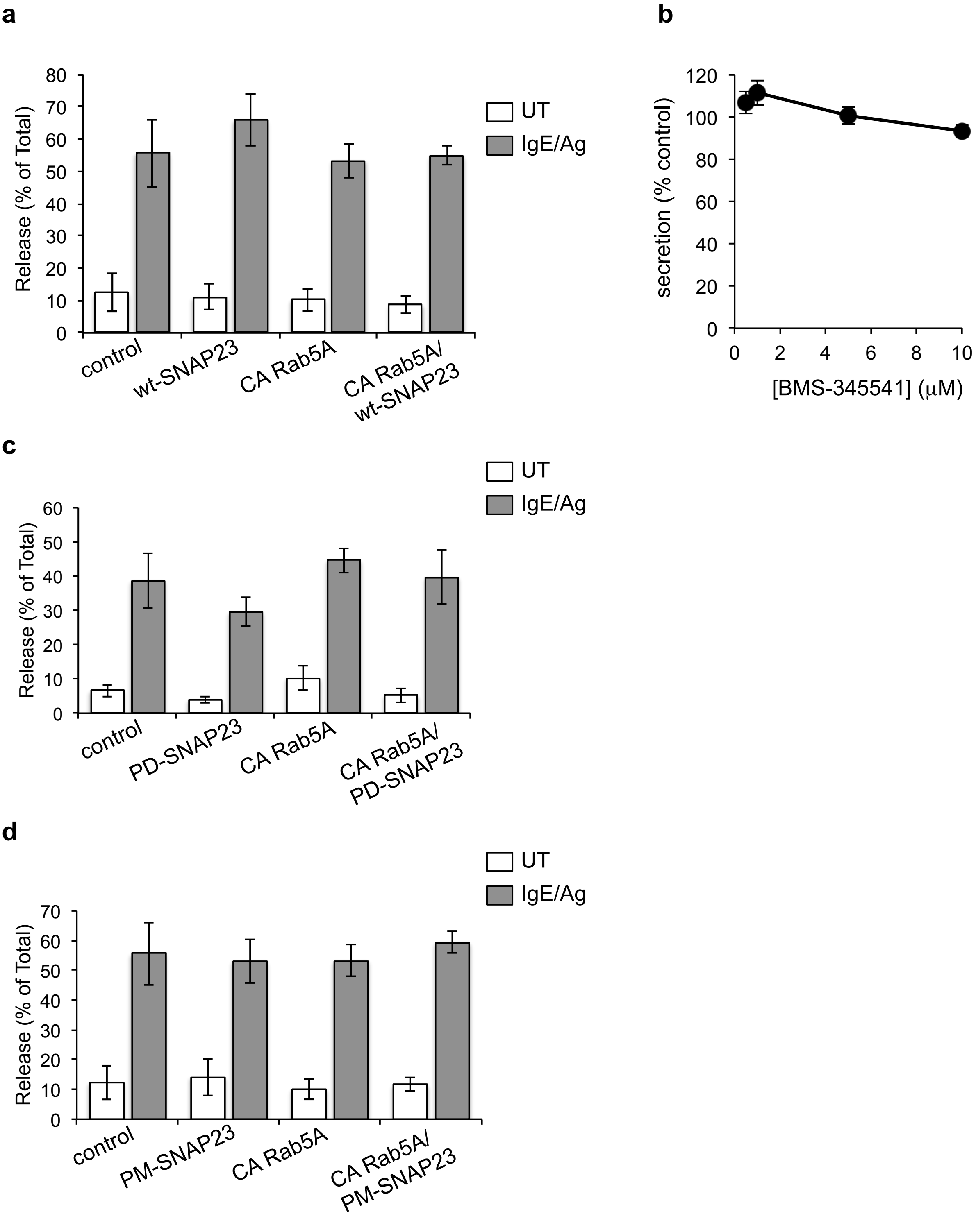

Supplementary Figure S1: Overexpression of SNAP23 or SNAP23 mutants does not influence secretion. (**a, c, d**) RBL cells were transiently co-transfected with 15 μg NPY-mRFP and either 15 μg pEGFP and 15 μg empty pcDNA3 (control), or 15 μg pEGFP and 15 μg of either HA-wt-SNAP23, HA-PD-SNAP23 or HA-PM-SNAP23, as indicated, or pEGFP-CA Rab5A and 15μg of empty pcDNA3, or 15 μg pEGFP-CA Rab5A and 15 μg of either HA-wt-SNAP23, HA-PD-SNAP23 or HA-PM-SNAP23 as indicated. Cells were sensitized with 1 μg/ml IgE. Twenty-four hours later cells were either left untreated (UT) or triggered with 50 ng/ml DNP-HSA (Ag) for 30 minutes. Release of NPY-mRFP was measured as previously described[36] and is presented as percentage of total. Data are means ± SEM of three independent experiments. (**b**) IgE-sensitized RBL cells were incubated with the indicated concentrations of BMS-345541 for 20 minutes at 37°C. Cells were subsequently triggered with 50 ng/ml DNP-HSA for 30 minutes. Release of endogenous β-hexosaminidase was measured as previously described[36] and is presented as percentage of total. Data are means ± SEM of two independent experiments.

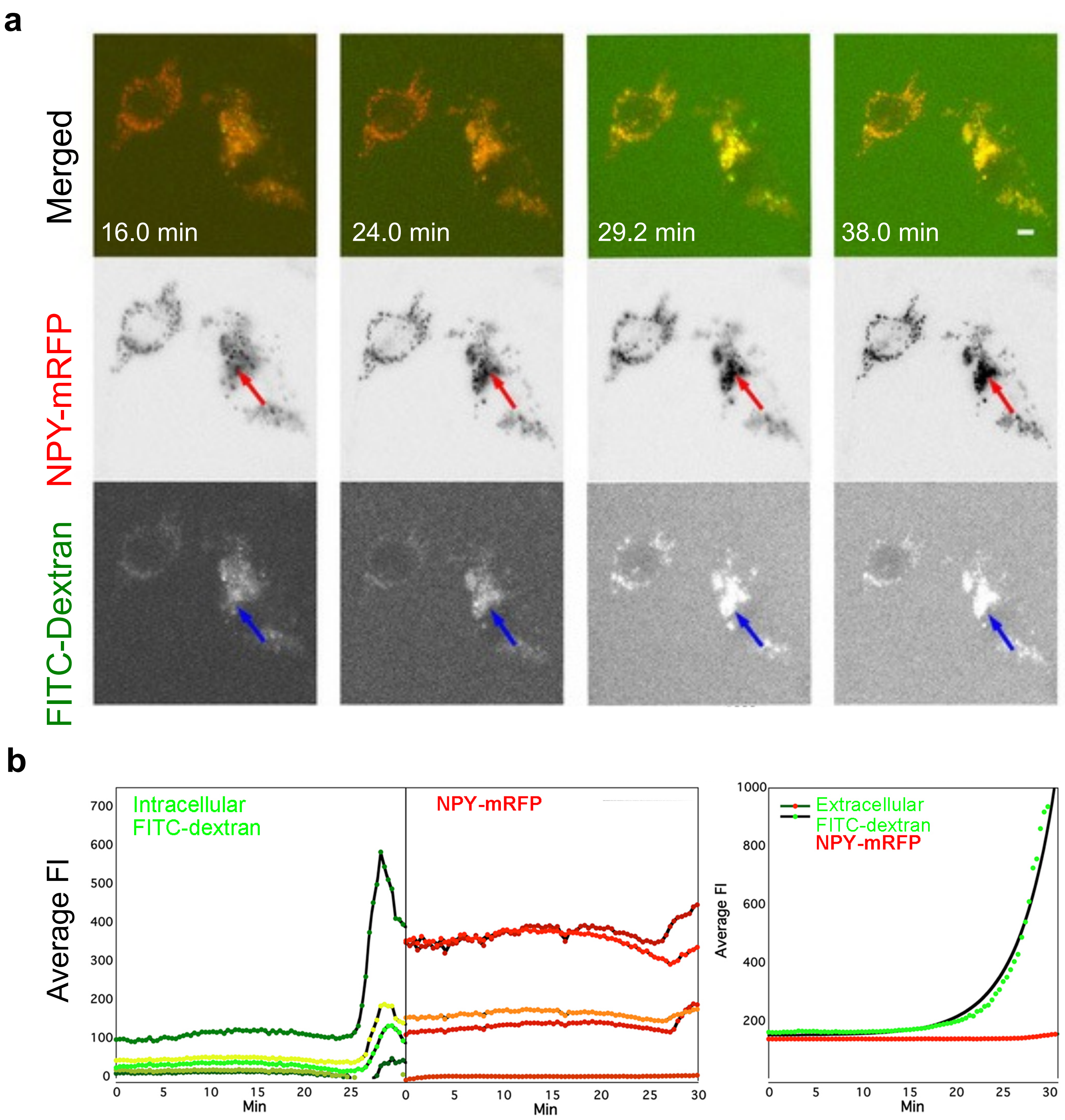

Supplementary Figure S2: Exocytotic events imaged in RBL-2H3 cells transfected with NPY-mRFP and pSilencer and loaded with FITC-dextran and stimulated with Ag. (**a**) RBL cells were co-transfected with 15 µg NPY-mRFP and 30 µg of pSilencer. Cells were sensitized with 1 µg/ml of IgE after loading with FITC-dextran (1 mg/ml) for 48 h. Cells were triggered by 50 ng/ml of DNP-HSA (Ag) and visualized by time-lapse fluorescence microscopy as described in the Materials and Methods. Designated times in the images are minutes after triggering with Ag. In (**a**), red and blue arrows point to SGs. Bars = 5 µm. **(b)** Quantitative analysis of the experiment shown in A. Each line in the left and middle graphs is the average fluorescence of a region of interest (ROI) over a single cell. The right hand graph is the fluorescence intensity in the medium. The black line is the simulated fitting based on an exponential first order reaction.

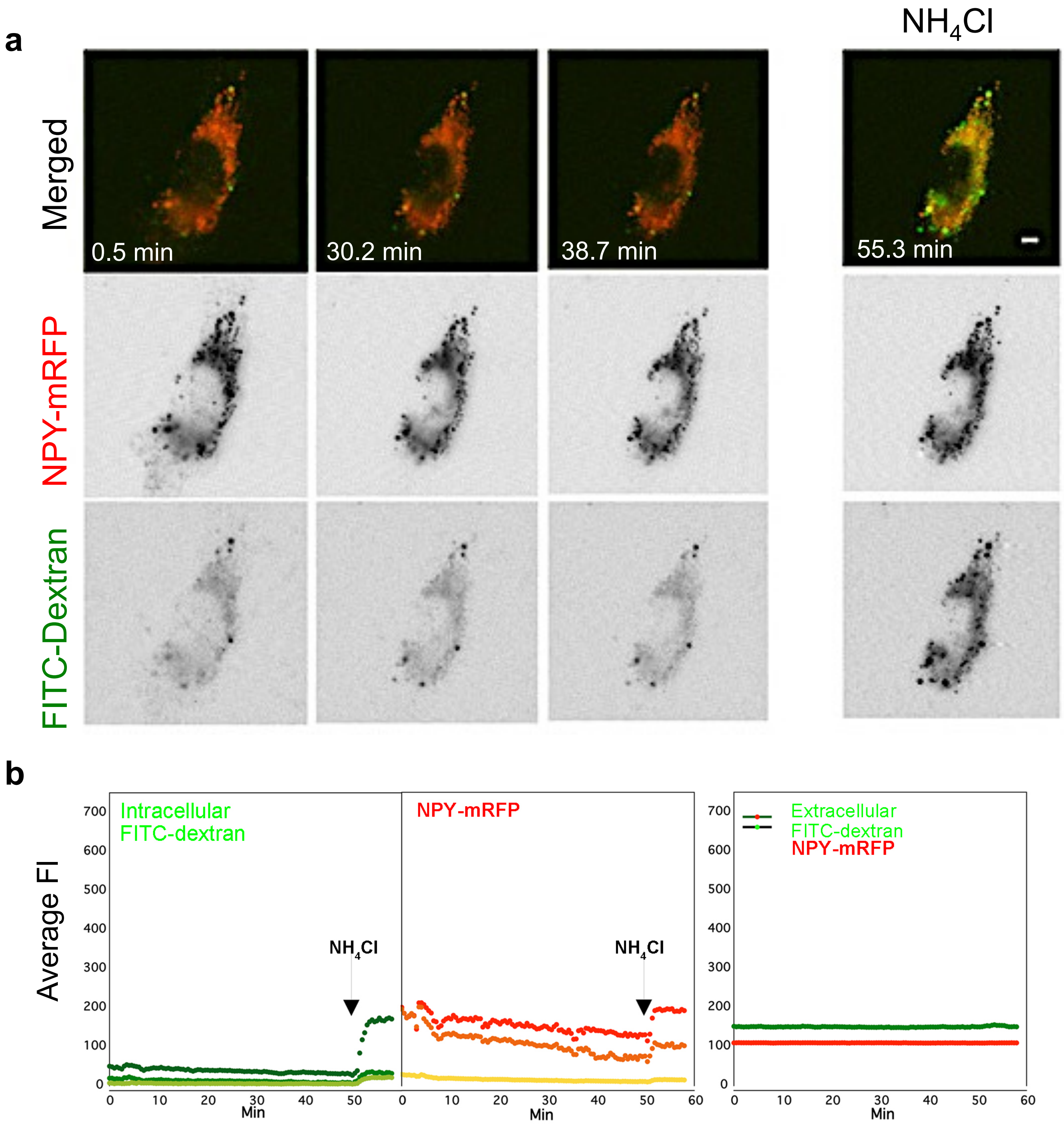

Supplementary Figure S3: Exocytotic events imaged in RBL-2H3 cells transfected with NPY-mRFP and shRab5A/B/C and loaded with FITC-dextran and stimulated with Ag. (**a**) RBL cells were co-transfected with 15 µg NPY-mRFP and 15 µg of shRab5A and 15 µg of shRab5B/C. Cells were sensitized with 1 µg/ml of IgE after loading with FITC-dextran (1 mg/ml) for 48 h. Cells were triggered by 50 ng/ml of DNP-HSA (Ag) and visualized by time-lapse fluorescence microscopy as described in the Materials and Methods. Designated times in the images are minutes after triggering with Ag. (**b**) Analysis is same as in Figure S2. The arrow points to the time of NH_4Cl (20 mM) addition. Bars = 5 µm.

Supplementary Videos

Video 1. Exocytotic events imaged in RBL-2H3 cells transfected with NPY-mRFP and pSilencer and loaded with FITC-dextran and stimulated with Ag. Ag was added at *t*=0. The video was taken using a Leica Sp5 laser scanning confocal microscope. Images were captured at 5 sec intervals.

Video 2. Exocytotic events imaged in RBL-2H3 cells transfected with NPY-mRFP and pSilencer and loaded with FITC-dextran and stimulated with Ag. Ag was added at *t*=0. The video was taken using a Zeiss LSM Pascal microscope. Images were captured at 30 sec intervals.

Video 3. Exocytotic events imaged in RBL-2H3 cells transfected with mStr-CA Rab5A and loaded with FITC-dextran and stimulated with Ag. Ag was added at *t*=0. The video was taken using a Zeiss LSM 800 microscope. Images were captured at 5 sec intervals.

Video 4. Exocytotic events imaged in RBL-2H3 cells transfected with mStr-CA Rab5A and HA-wt-SNAP23 and loaded with FITC-dextran and stimulated with Ag. Ag was added at *t*=0. The video was taken using a Zeiss LSM 800 microscope. Images were captured at 5 sec intervals.

Video 5. Exocytotic events imaged in RBL-2H3 cells transfected with NPY-mRFP and Rab5A/B/C-targeting shRNA and loaded with FITC-dextran and stimulated with Ag. Ag was added at *t*=0. The video was taken using a Leica Sp5 laser scanning confocal microscope. Images were captured at 5 sec intervals.

Video 6. Exocytotic events imaged in RBL-2H3 cells transfected with NPY-mRFP and Rab5A/B/C-targeting shRNA and loaded with FITC-dextran and stimulated with Ag. Ag was added at *t*=0. The video was taken using a Zeiss LSM Pascal microscope. Images were captured at 30 sec intervals.
